# What Drives Health Professionals to Tweet About #HPVvaccine? Identifying Strategies for Effective Communication

**DOI:** 10.5888/pcd15.170320

**Published:** 2018-02-22

**Authors:** Philip M. Massey, Alex Budenz, Amy Leader, Kara Fisher, Ann C. Klassen, Elad Yom-Tov

**Affiliations:** 1 Department of Community Health and Prevention, Dornsife School of Public Health, Drexel University, Philadelphia, Pennsylvania; 2 Division of Population Science, Department of Medical Oncology, Thomas Jefferson University, Philadelphia, Pennsylvania; 3 Association of American Medical Colleges, Washington, DC; 4 Microsoft Research Israel, Herzeliya, Israel

## Abstract

**Introduction:**

We conducted this study to quantify how health professionals use Twitter to communicate about the human papillomavirus (HPV) vaccine.

**Methods:**

We collected 193,379 tweets from August 2014 through July 2015 that contained key words related to HPV vaccine. We classified all tweets on the basis of user, audience, sentiment, content, and vaccine characteristic to examine 3 groups of tweets: 1) those sent by health professionals, 2) those intended for parents, and 3) those sent by health professionals and intended for parents. For each group, we identified the 7-day period in our sample with the most number of tweets (spikes) to report content.

**Results:**

Of the 193,379 tweets, 20,451 tweets were from health professionals; 16,867 tweets were intended for parents; and 1,233 tweets overlapped both groups. The content of each spike varied per group. The largest spike in tweets from health professionals (n = 851) focused on communicating recently published scientific evidence. Most tweets were positive and were about resources and boys. The largest spike in tweets intended for parents (n = 1,043) centered on a national awareness day and were about resources, personal experiences, boys, and girls. The largest spike in tweets from health professionals to parents (n = 89) was in January and centered on an event hosted on Twitter that focused on cervical cancer awareness month.

**Conclusion:**

Understanding drivers of tweet spikes may help shape future communication and outreach. As more parents use social media to obtain health information, health professionals and organizations can leverage awareness events and personalize messages to maximize potential reach and parent engagement.

## Introduction

Approximately 80 million people in the United States, or approximately 1 in 4, are infected with human papillomavirus (HPV), and 14 million new cases of HPV occur each year (1). Long-term infection with certain types of HPV can cause cancer and genital warts, and a major public health advancement has been the development of vaccines that protect against these cancer-related strains of HPV (1). The vaccine is recommended for girls and boys (2), and updated 2016 guidelines recommend that a 2-dose series be administered 6 to 12 months apart (3).

Although vaccine rates have increased over the past 10 years, national rates continue to fall short of the Healthy People 2020 goal of 80% HPV vaccine coverage (4). Understanding the complex barriers surrounding HPV vaccine acceptance, particularly related to parent resistance, is key to strengthening vaccine uptake among adolescents (5). A key predictor of HPV vaccine uptake is a health professional’s recommendation (6,7). Although the Centers for Disease Control and Prevention provides guidelines for vaccine administration, one study found that some health professionals were more likely to strongly recommend the vaccine to older children than to younger children, and to girls than to boys (8). As more parents and health professionals are turning to online resources for health information and communication, there is an opportunity to address barriers and misinformation through strategic information dissemination and communication (9,10).

Social media, and particularly Twitter, is an online resource that many use to look for health information and to communicate about their health and health care experiences (11). There has been a recent increase in vaccine resources that parents can access through social media (12); however, many of these resources disseminate false information, speak out against medical professionals, and perpetuate fear of vaccines (13). To address health consumers’ growing use of online health information, various reports underscore the importance for health professionals to incorporate digital communication into their practice (14,15).

Given that a major driver of HPV vaccine uptake is a health professional’s recommendation and that more parents are turning to online resources for health information, we sought to examine how these topics are discussed on Twitter, particularly the communication pattern of health professionals related to the HPV vaccine on Twitter. Furthermore, drawing from research that examined the predictive nature of communication spikes on Twitter — specifically how online spikes may be associated with offline behaviors — we conducted a subanalysis of communication spikes to both describe current patterns and inform future use of this communication channel for preventive health care, in particular the HPV vaccine (16).

This study adds to a growing body of literature quantifying the use of Twitter to communicate health information (17–20), with a focus on health professionals and parents. The purpose of this study was to characterize and quantify 3 types of Twitter messages related to the HPV vaccine: 1) tweets sent by health professionals, 2) tweets intended for a parent audience, and 3) tweets sent by health professionals and intended for a parent audience.

## Methods

We used data mining software to access the Twitter Search API to collect prospective data (21). We defined our inclusion criteria as tweets that contained any of our 5 search terms related to the HPV vaccine (“HPV,” “HPV vaccine,” “HPV shot,” “Gardasil,” and “Cervarix”) as well as the 5 corresponding hashtags. Our final data set contained 193,379 English-only tweets collected from August 1, 2014, through July 31, 2015, and included both original tweets and retweets; that is, whether the tweet was original or not. If a user’s tweet contained any of our key words, it was included in the final sample. We developed a codebook to analyze the content of the tweet text for HPV vaccine sentiment and characteristics and coded a subsample of tweets by hand (n = 1,470). We used the coded tweets to build classifiers using supervised machine-learning to classify our entire sample. Classifiers used in this study, along with the corresponding accuracy as measured by the area under the receiver operating characteristic curve (AUC), included the following: type of user (AUC = 0.75), target audience (AUC = 0.95), vaccine sentiment (AUC = 0.92), content (AUC = 0.72), side effects (AUC = 0.74), prevention/protection (AUC = 0.77), risk/prevalence (AUC = 0.88), men/boys (AUC = 0.92), and women/girls (AUC = 0.92). Our full methods and classification process are described elsewhere (22).

We operationalized our 3 subsample tweet types using the following study variables: 1) type of user = health professional; 2) target audience = parent; and 3) type of user = health professional AND target audience = parent (consisting of an overlap of the first 2 categories). For each type of tweet, we identified the period with the highest volume of tweets (or “spikes”) during our study. To examine activity surrounding these spikes, we considered 3 days before and after each spike, totaling a 7-day period.

For each spike we classified the content of the tweets, including type of tweet, tweet sentiment, tweet content, and vaccine characteristics (Box 1). The purpose of these analyses was to ascertain the nature of HPV messaging during periods with the highest volume of posts. We also quantified the potential reach of the tweets, on the basis of the average and median number of followers and engagement features for each tweet (ie, hashtags and mentions), because research has used these features as a measure of influence on social media (23,24). For a sample of tweets (approximately 10% of each spike), we examined links to websites that were included in the tweets by opening the link in a web browser. Finally, because a large number of tweets sent by health professionals were associated with a recently published article, we also examined a sample of web links from the second and third largest spikes for this group. We examined additional spikes only for health professionals to confirm preliminary findings. To measure significant differences across spikes, we used χ^2^ tests for categorical variables, analysis of variance for continuous variables, and the Kruskal–Wallis nonparametric test for medians. The study protocol was deemed exempt, and all procedures were approved by the Drexel University Institutional Review Board.

**Box 1. Names and Description of Study Variables, Study of Twitter Messages Related to the Human Papillomavirus (HPV) Vaccine, August 2014–July 2015**

**Table:** 

**Type of tweet**
Health professional user: tweets posted by health professionals
Parent audience: tweets posted with content for parents, messages intended to reach parents
Health professional to parent: tweets posted by health professionals for a parent audience
**Tweet content**
Resource/information: objective information about the HPV vaccine with supporting source
Personal experience/opinion: story or opinion about HPV, no factual sources
Joke or parody: humorous/satirical statement about HPV
Other: tweet does not satisfy the above categories
**Tweet characteristic**
Side effects: mention of side effects caused by the HPV vaccine
Prevention/protection: describes how HPV vaccine will protect or prevent negative health outcomes
Risk/prevalence of HPV: details risk of contracting HPV or the prevalence of HPV
Men/boys: tweet refers to men or boys along with HPV
Women/girls: refers to women or girls along with HPV
**Tweet sentiment**
Positive: supportive messages about the HPV vaccine and encourages its uptake
Negative: disparaging messages about the HPV vaccine and/or discourages it uptake
Neutral: no opinions about vaccine, facts repeated
No mention: HPV vaccine is not mentioned

## Results

Among tweets sent by health professionals (n = 20,451), the largest number of tweets were sent on April 13, 2015 (n = 381) (Figure). Among tweets intended for a parent audience (n = 16,867), the largest number of tweets were sent on February 4, 2015 (n = 494). Finally, among tweets sent by health professionals and intended for a parent audience (n = 1,233), the greatest number of tweets occurred on January 22, 2015 (n = 39).

**Figure:**
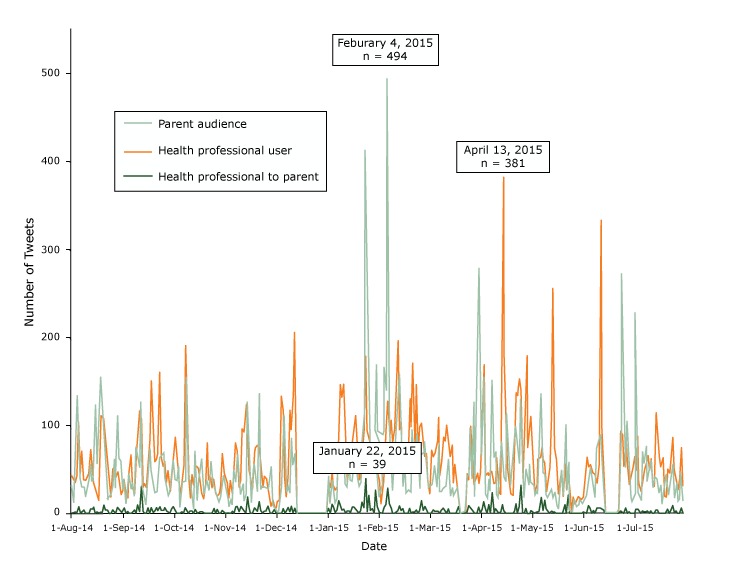
Number of tweets, by group subsample over time, indicating the day with the most tweets for each group, study of twitter messages related to the human papillomavirus vaccine, August 2014-July 2015. **Table:** 

Date Created	Type of Tweet, No.		
	Health Professional User	Health Professional to Parent	Parent Audience
1-Aug-14	43	1	32
2-Aug-14	38	1	14
3-Aug-14	35	2	62
4-Aug-14	43	0	133
5-Aug-14	103	8	63
6-Aug-14	46	1	38
7-Aug-14	71	2	30
8-Aug-14	39	4	30
9-Aug-14	38	0	20
10-Aug-14	41	1	31
11-Aug-14	46	1	39
12-Aug-14	72	5	56
13-Aug-14	50	5	36
14-Aug-14	33	7	39
15-Aug-14	25	0	122
16-Aug-14	19	2	57
17-Aug-14	15	1	76
18-Aug-14	111	2	154
19-Aug-14	109	2	107
20-Aug-14	108	10	82
21-Aug-14	73	4	57
22-Aug-14	57	4	16
23-Aug-14	39	1	19
24-Aug-14	19	0	23
25-Aug-14	50	4	31
26-Aug-14	53	3	62
27-Aug-14	41	0	28
28-Aug-14	47	7	110
29-Aug-14	34	5	63
30-Aug-14	22	2	59
31-Aug-14	25	2	28
1-Sep-14	38	6	39
2-Sep-14	24	1	11
3-Sep-14	18	2	36
4-Sep-14	50	0	46
5-Sep-14	66	0	27
6-Sep-14	28	2	36
7-Sep-14	23	4	20
8-Sep-14	61	1	76
9-Sep-14	97	10	68
10-Sep-14	116	3	53
11-Sep-14	107	31	126
12-Sep-14	30	3	37
13-Sep-14	33	0	21
14-Sep-14	30	1	10
15-Sep-14	62	6	47
16-Sep-14	86	1	73
17-Sep-14	150	6	49
18-Sep-14	106	2	30
19-Sep-14	59	3	24
20-Sep-14	62	6	25
21-Sep-14	68	4	34
22-Sep-14	160	7	63
23-Sep-14	63	4	68
24-Sep-14	54	4	70
25-Sep-14	59	6	40
26-Sep-14	36	3	29
27-Sep-14	48	2	20
28-Sep-14	25	0	23
29-Sep-14	39	5	36
30-Sep-14	54	7	61
1-Oct-14	87	4	39
2-Oct-14	61	0	37
3-Oct-14	45	1	54
4-Oct-14	23	1	8
5-Oct-14	30	2	28
6-Oct-14	54	1	36
7-Oct-14	190	6	37
8-Oct-14	145	4	156
9-Oct-14	40	1	38
10-Oct-14	18	3	37
11-Oct-14	44	3	26
12-Oct-14	33	1	6
13-Oct-14	52	4	70
14-Oct-14	53	0	15
15-Oct-14	51	1	36
16-Oct-14	35	2	38
17-Oct-14	23	2	61
18-Oct-14	17	1	21
19-Oct-14	29	0	33
20-Oct-14	79	0	38
21-Oct-14	39	1	27
22-Oct-14	40	1	60
23-Oct-14	30	1	42
24-Oct-14	38	5	30
25-Oct-14	23	0	28
26-Oct-14	20	4	18
27-Oct-14	22	0	13
28-Oct-14	30	1	17
29-Oct-14	68	3	26
30-Oct-14	46	2	43
31-Oct-14	42	0	28
1-Nov-14	33	1	21
2-Nov-14	18	0	8
3-Nov-14	37	0	16
4-Nov-14	23	2	11
5-Nov-14	62	0	49
6-Nov-14	80	7	35
7-Nov-14	69	8	68
8-Nov-14	30	3	38
9-Nov-14	38	7	21
10-Nov-14	96	5	34
11-Nov-14	93	3	16
12-Nov-14	122	2	23
13-Nov-14	98	18	126
14-Nov-14	50	7	62
15-Nov-14	42	0	14
16-Nov-14	28	1	50
17-Nov-14	67	6	57
18-Nov-14	75	4	39
19-Nov-14	77	3	26
20-Nov-14	75	13	136
21-Nov-14	56	3	28
22-Nov-14	30	0	32
23-Nov-14	42	3	30
24-Nov-14	40	3	29
25-Nov-14	33	8	28
26-Nov-14	19	2	45
27-Nov-14	9	1	53
28-Nov-14	15	2	9
29-Nov-14	7	4	9
30-Nov-14	7	0	3
1-Dec-14	14	0	3
2-Dec-14	15	1	17
3-Dec-14	132	0	27
4-Dec-14	116	5	62
5-Dec-14	43	1	111
6-Dec-14	20	2	20
7-Dec-14	49	6	30
8-Dec-14	117	3	23
9-Dec-14	96	8	86
10-Dec-14	134	2	55
11-Dec-14	205	5	68
12-Dec-14	0	0	0
13-Dec-14	0	0	0
14-Dec-14	0	0	0
15-Dec-14	0	0	0
16-Dec-14	0	0	0
17-Dec-14	0	0	0
18-Dec-14	0	0	0
19-Dec-14	0	0	0
20-Dec-14	0	0	0
21-Dec-14	0	0	0
22-Dec-14	0	0	0
23-Dec-14	0	0	0
24-Dec-14	0	0	0
25-Dec-14	0	0	0
26-Dec-14	0	0	0
27-Dec-14	0	0	0
28-Dec-14	0	0	0
29-Dec-14	0	0	0
30-Dec-14	0	0	0
31-Dec-14	36	5	42
1-Jan-15	32	1	11
2-Jan-15	49	10	37
3-Jan-15	33	8	31
4-Jan-15	27	2	12
5-Jan-15	30	4	17
6-Jan-15	89	14	69
7-Jan-15	146	8	54
8-Jan-15	132	4	89
9-Jan-15	147	5	91
10-Jan-15	65	1	34
11-Jan-15	36	3	30
12-Jan-15	62	3	60
13-Jan-15	72	2	39
14-Jan-15	92	7	37
15-Jan-15	84	7	90
16-Jan-15	111	5	81
17-Jan-15	68	3	41
18-Jan-15	39	0	30
19-Jan-15	43	4	36
20-Jan-15	90	12	89
21-Jan-15	75	7	106
22-Jan-15	178	39	412
23-Jan-15	71	3	76
24-Jan-15	58	20	275
25-Jan-15	78	4	71
26-Jan-15	76	5	68
27-Jan-15	76	2	75
28-Jan-15	92	26	171
29-Jan-15	45	8	48
30-Jan-15	37	2	19
31-Jan-15	12	1	23
1-Feb-15	22	1	35
2-Feb-15	62	7	165
3-Feb-15	98	10	139
4-Feb-15	127	28	494
5-Feb-15	83	11	92
6-Feb-15	105	7	95
7-Feb-15	67	1	25
8-Feb-15	100	0	63
9-Feb-15	165	2	51
10-Feb-15	196	3	66
11-Feb-15	90	8	158
12-Feb-15	102	3	107
13-Feb-15	44	5	32
14-Feb-15	41	1	50
15-Feb-15	58	0	22
16-Feb-15	57	1	27
17-Feb-15	129	5	24
18-Feb-15	99	0	24
19-Feb-15	170	2	95
20-Feb-15	74	2	54
21-Feb-15	146	4	91
22-Feb-15	70	1	60
23-Feb-15	98	3	27
24-Feb-15	102	14	66
25-Feb-15	80	4	67
26-Feb-15	65	8	82
27-Feb-15	68	4	62
28-Feb-15	35	1	29
1-Mar-15	23	0	29
2-Mar-15	70	2	33
3-Mar-15	65	4	39
4-Mar-15	67	3	54
5-Mar-15	80	3	27
6-Mar-15	109	3	80
7-Mar-15	44	0	24
8-Mar-15	58	4	25
9-Mar-15	85	6	29
10-Mar-15	83	3	30
11-Mar-15	100	1	35
12-Mar-15	78	5	62
13-Mar-15	65	0	19
14-Mar-15	77	1	31
15-Mar-15	35	0	21
16-Mar-15	55	1	25
17-Mar-15	33	2	32
18-Mar-15	16	8	18
19-Mar-15	0	0	0
20-Mar-15	0	0	0
21-Mar-15	0	0	0
22-Mar-15	0	0	0
23-Mar-15	43	2	15
24-Mar-15	40	9	60
25-Mar-15	97	7	87
26-Mar-15	77	4	37
27-Mar-15	34	2	126
28-Mar-15	39	0	19
29-Mar-15	44	1	109
30-Mar-15	35	7	278
31-Mar-15	50	2	44
1-Apr-15	78	4	182
2-Apr-15	167	13	130
3-Apr-15	43	5	23
4-Apr-15	46	1	17
5-Apr-15	44	10	40
6-Apr-15	47	0	25
7-Apr-15	47	23	150
8-Apr-15	34	2	63
9-Apr-15	35	5	70
10-Apr-15	61	2	21
11-Apr-15	24	0	18
12-Apr-15	22	2	33
13-Apr-15	381	10	83
14-Apr-15	186	3	42
15-Apr-15	132	1	36
16-Apr-15	52	0	114
17-Apr-15	44	1	74
18-Apr-15	22	0	30
19-Apr-15	21	1	32
20-Apr-15	79	2	69
21-Apr-15	136	11	99
22-Apr-15	135	1	90
23-Apr-15	153	8	57
24-Apr-15	133	32	141
25-Apr-15	41	2	26
26-Apr-15	50	0	17
27-Apr-15	178	5	50
28-Apr-15	75	1	32
29-Apr-15	102	1	57
30-Apr-15	110	3	36
1-May–15	76	2	33
2-May–15	25	0	21
3-May–15	32	2	25
4-May–15	63	2	23
5-May–15	63	3	31
6-May–15	75	18	136
7-May–15	62	4	46
8-May–15	56	15	45
9-May–15	26	5	20
10-May–15	37	3	18
11-May–15	49	1	25
12-May–15	111	3	27
13-May–15	255	2	19
14-May–15	74	2	24
15-May–15	61	0	9
16-May–15	23	0	4
17-May–15	26	0	27
18-May–15	43	1	49
19-May–15	20	1	19
20-May–15	54	10	101
21-May–15	27	1	9
22-May–15	43	21	59
23-May–15	5	0	4
24-May–15	3	1	2
25-May–15	18	0	4
26-May–15	11	0	16
27-May–15	14	0	8
28-May–15	19	1	9
29-May–15	17	0	17
30-May–15	16	0	11
31-May–15	18	2	14
1-Jun-15	33	1	17
2-Jun-15	63	1	20
3-Jun-15	53	9	34
4-Jun-15	54	6	54
5-Jun-15	49	0	34
6-Jun-15	46	3	21
7-Jun-15	45	1	16
8-Jun-15	35	6	72
9-Jun-15	53	6	89
10-Jun-15	332	6	90
11-Jun-15	102	5	51
12-Jun-15	38	4	32
13-Jun-15	0	0	0
14-Jun-15	0	0	0
15-Jun-15	0	0	0
16-Jun-15	0	0	0
17-Jun-15	0	0	0
18-Jun-15	0	0	0
19-Jun-15	0	0	0
20-Jun-15	0	0	0
21-Jun-15	5	0	4
22-Jun-15	93	3	42
23-Jun-15	88	1	270
24-Jun-15	54	0	95
25-Jun-15	81	6	49
26-Jun-15	47	0	105
27-Jun-15	34	2	68
28-Jun-15	36	1	19
29-Jun-15	45	1	14
30-Jun-15	64	0	23
1-Jul-15	50	5	227
2-Jul-15	86	5	29
3-Jul-15	36	4	20
4-Jul-15	26	0	14
5-Jul-15	32	3	69
6-Jul-15	48	2	53
7-Jul-15	51	2	77
8-Jul-15	64	3	36
9-Jul-15	50	1	60
10-Jul-15	62	3	59
11-Jul-15	30	1	30
12-Jul-15	31	1	36
13-Jul-15	114	4	41
14-Jul-15	100	4	39
15-Jul-15	64	1	21
16-Jul-15	53	0	33
17-Jul-15	63	1	10
18-Jul-15	21	0	25
19-Jul-15	19	1	44
20-Jul-15	49	5	32
21-Jul-15	47	0	22
22-Jul-15	72	4	32
23-Jul-15	86	9	57
24-Jul-15	51	2	16
25-Jul-15	38	3	36
26-Jul-15	28	0	14
27-Jul-15	41	1	15
28-Jul-15	75	6	47
29-Jul-15	32	1	13
Total	20,821	1,245	16,971
Percentage	5.98		7.34

In both spikes in which tweets were intended for a parent audience, almost all tweets were retweets (97.1% for parent audience and 89.9% for health professional to parent audience), compared with the health professional spike where approximately half of the tweets were retweets (50.1%) (Table).

**Table:** Tweet Characteristics, by Spike, Compared With Full Sample of Tweets, Study of Twitter Messages Related to the Human Papillomavirus Vaccine, August 2014–July 2015^a^

Characteristic	Type of Tweet			Full Sample of Tweets, August 2014–July 2015 (n = 193,379)
	Health Professional User (n = 851)	Parent Audience (n = 1,043)	Health Professional to Parent Audience (n = 89)	
**Spike range, dates**	4/10/15–4/16/15	2/1/15–2/7/15	1/19–1/25/15	**—**
**Day with most tweets**	Monday 4/13/15	Wednesday 2/4/15	Thursday 1/22/15	Wednesday 4/29/15
**No. of followers per tweet**				
Mean	1,015	1,501	888	1,493
Median	396	454	395	440
**Original/retweet, no (%)^b^**				
Tweet is original	425 (49.9)	30 (2.9)	9 (10.1)	108,974 (56.4)
Tweet is a retweet	426 (50.1)	1,013 (97.1)	80 (89.9)	84,405 (43.6)
**Links (URL) per Tweet, no (%)^b^**				
0	152 (17.9)	1,035 (99.2)	73 (82.0)	55,320 (28.6)
1	564 (66.3)	7 (0.7)	10 (11.2)	116,701 (60.4)
≥2	135 (15.9)	1 (0.1)	6 (6.7)	21,358 (11.0)
**Mentions (@) per Tweet, no (%)^b^**				
0	346 (40.7)	23 (2.2)	7 (7.9)	81,330 (42.1)
1	385 (45.2)	711 (68.2)	48 (53.9)	79,653 (41.2)
2 or more	120 (14.1)	309 (29.6)	34 (38.2)	32,396 (16.7)
**Hashtags (#) per Tweet, no (%)^b^**				
0	486 (57.1)	429 (41.1)	14 (15.7)	106,413 (55.0)
1	194 (22.8)	375 (36.0)	29 (32.6)	36,892 (19.1)
≥2	171 (20.1)	239 (22.9)	46 (51.7)	50,074 (25.9)
**Spike sentiment, no (%)^b^**				
Positive	567 (66.6)	491 (47.1)	32 (36.0)	75,393 (39.0)
Negative	99 (11.6)	199 (19.1)	4 (4.5)	48,940 (25.3)
Neutral	102 (12.0)	133 (12.8)	8 (9.0)	25,110 (13.0)
No mention of vaccine	83 (9.8)	220 (21.1)	45 (50.6)	43,936 (22.7)
**Spike content, no (%)^b^**				
Resource/information	554 (65.1)	365 (35.0)	41 (46.1)	98,484 (50.9)
Personal experience/opinion	290 (34.1)	625 (59.9)	46 (51.7)	91,042 (47.1)
Joke or parody	3 (0.4)	53 (5.1)	2 (2.2)	3,428 (17.7)
Other	4 (0.5)	0	0	425 (0.2)
**Spike vaccine characteristics, no (%)^c^**				
Side effects^b^	91 (10.7)	177 (17.0)	11 (12.4)	42,989 (22.2)
Prevention/protection^b^	302 (35.5)	125 (12.0)	5 (5.6)	36,591 (18.9)
Risk/prevalence^b^	88 (10.3)	183 (17.5)	14 (15.7)	21,529 (11.1)
Men/boys^b^	526 (61.8)	94 (9.0)	16 (18.0)	18,971 (9.8)
Women/girls^d^	83 (9.8)	138 (13.2)	8 (9.0)	21,407 (11.1)

a To measure significant differences across spikes, we used χ^2^ tests for categorical variables, analysis of variance for continuous variables, and the Kruskal–Wallis nonparametric test for medians.

b *P* < .001.

c Not mutually exclusive.

d *P* = .04.

The use of twitter engagement features, including links, mentions (@), and hashtags (#), also differed across the 3 spikes. In the health professional spike, most tweets included one link (66.3%), whereas most tweets in the other spikes did not include a link. Nearly all tweets in both parent audience spikes included at least one mention (97.7% for parent audience and 92.1% for health professional to parent audience). Among the 3 spikes, the health professional spike had the highest percentage of tweets that did not include a hashtag (57.1%) (Table).

The largest number of tweets sent by health professionals was from April 10 to April 16, 2015 (n = 851), and 66.6% of these tweets were classified as positive. In addition, 65.1% discussed resources or information, and 61.8% of tweets talked about men or boys. After examining a 10% sample of tweets from the spike, we found that many of the web links provided in the tweets referenced a study published in *Cancer* that conducted a cost-effectiveness analysis of HPV vaccination for boys (25).

Many of the tweets in the 2 additional spikes also focused on recently published studies on HPV vaccine research. The content of the second largest health professional spike, in June 2015, focused on an article published in *Lancet Oncology* that examined the efficacy of fewer doses of the HPV vaccine (26). Similarly, the content of the third largest health professional spike, in May 2015, was associated with an article published in *BMJ* that examined direct and indirect benefits to males when only girls are immunized (27).

Among tweets intended for a parent audience, the greatest number of tweets were sent from February 1 to February 7, 2015 (n = 1,043), and the greatest sentiment expressed in the parent audience spike was positive (47.1%). Nearly 60% of tweets in this spike discussed personal experiences or expressed opinions, followed by 35.0% of tweets discussing resources and information. The vaccine characteristics discussed in these tweets focused on risk and prevalence (17.5%), side effects (17.0%), and women or girls (13.2%). After further reviewing tweet content in this spike, we determined that many of the tweets intended for parent audiences corresponded with World Cancer Day, an annual awareness day taking place on February 4.

The largest spike in tweets from health professionals to a parent audience occurred from January 19, 2015, to January 25, 2015 (n = 89). Approximately 50% of tweets did not mention the HPV vaccine, followed by 36% of tweets having a positive sentiment toward the vaccine. The overall content of this spike was split nearly evenly between personal experiences/opinions (52%), and resources/information (46%). Examination of tweets in this spike indicated that many of the tweets were associated with a specific Twitter chat event focused on the hashtags #hpv and #teenhealth. The event took place during cervical cancer awareness month, occurring annually during January. Box 2 provides sample tweets from each of the spikes.

**Box 2. Sample Tweets from Each of the Largest Spikes, by Type of Tweet, Study of Twitter Messages Related to the Human Papillomavirus (HPV) Vaccine, August 2014–July 2015**

**Table:** 

**Health professional user spike**
RT @globeandmail: Vaccinating boys against HPV could cut health-care costs, study suggests http://t.co/kHKeVRSr7o
RT @CBCNews: HPV vaccination for boys needs more awareness: oncologist & MP http://t.co/GortY8bIML http://t.co/vmB50Tb3ql
@FSSWorldIssues HPV Vaccination for boys can be a money saver! http://t.co/Uivs5r9ERa
**Parent audience spike**
RT @CDCSTD: #Parents: You don't open the door to #sex w/ #HPV #vaccine. You close the door to #cancer! http://t.co/a4iDQqmwpA
@TheSocialCTV I trust the ones we got as a kid. Tried & true ones. However HPV seems like a corporate sponsored vaccine that I don’t trust.
RT @VaxCalc: HPV (Gardasil) and Hep-B are lifestyle vaccines; do infants and children really need these? #parenting
**Health professional to parent audience spike**
RT @jhforg: RT @iTwixie: Thank you, @JHForg for sponsoring this important #TeenHealth chat! #HPV
@iTwixie thank you and @wgfpa @DrMaryxxxxx @igpxxxxx and all of our other participants! it was great to talk about #hpv and #teenhealth
RT @nycHealthy: For #CervicalCancer Awareness Month, ask your child's doctor about the HPV vaccine. #VaccinateHPV http://t.co/Uu6dCARNQ3

When comparing the spike subsamples with the full sample of tweets, we found differences in many of the tweet characteristics (Box 2). The number of original tweets and retweets differed significantly between the full sample and spike subsamples (*P* < .001), as did the use of Twitter engagement features, including links, mentions, and hashtags (*P* < .001). In general, positive sentiment was more prevalent in the health professional spike (66.6%) and parent audience spike (47.1%) compared with the full sample (39.0%, *P* < .001). The percentage of tweets that included resources or information was higher in the health professional spike (65.1%) compared with the full sample (50.9%, *P* < .001), and a higher percentage of tweets in the parent audience spike included personal experiences or opinion (59.9%) compared with our full sample (47.1%, *P* < .001). One of the largest differences between the health professional spike and the full sample of tweets was the percentage of tweets that focused on men/boys (61.8% vs 9.8%, respectively, *P* < .001). Prevention/protection was also a vaccine characteristic that was more prevalent in the health professional spike (35.5%) compared with all tweets (18.9%, *P* < .001). Conversely, side effects were mentioned more frequently in our all tweet sample (22.2%) compared with each of the spikes, including the health professional spike (10.7%, *P* < .001).

## Discussion

Findings from our study suggest different drivers of spikes in Twitter communication for tweets sent by health professionals and for tweets intended for parents. Health professional Twitter activity spiked around the communication of scientific findings, namely disseminating and discussing new scientific evidence. Conversely, parents’ tweets centered most often on awareness campaigns and national awareness events.

Our findings also show the importance of retweets in creating spikes and generating tweet volume. In 2 of the 3 spike periods, nearly all of the tweets were retweets, a much higher percentage than in the full sample of tweets. These data suggest that behaviors during awareness days and national campaigns may differ from general use behaviors, and capitalizing on the “retweet” phenomenon during these spike events is worth further exploration and could lead to a heightened level of interest and engagement in the topic area (16).

For health professionals on Twitter, the dissemination of new scientific evidence was a key driver of Twitter activity. Furthermore, we found that many of the links in the tweets were links to news stories in the popular media that covered the publication, rather than the original publication. The use of links to news stories may be due to the ease of linking a popular news source to Twitter messages (eg, by clicking a “share on Twitter” button), as opposed to creating and embedding a URL in the tweet for the peer-reviewed publication. In addition, many peer-reviewed articles are only accessible if a subscription is paid, limiting the public’s access. Another benefit of linking to a popular news source may be that the reporting is more relevant to a lay audience. Future studies may consider the impact of disseminating a popular news source that reports on a new study versus the original peer-reviewed article of the study. This consideration is particularly relevant in an era marked by the proliferation of online fake news.

Tweets in the health professional spike also included far fewer hashtags than the other 2 spikes, which could lead to less exposure or engagement with a wider audience. One way users are exposed to tweets is that a user must follow another user to see their tweets. This way, if a health professional tweets about HPV vaccine, all of their followers would be exposed to the tweet content. However, additional avenues for exposure and engagement exist, including users searching for key words on Twitter or clicking on a hashtag that is a topic of interest. This avenue leads to indirect exposure, as a user would read a tweet about HPV vaccine from a user they do not follow. This indirect exposure strategy, which uses different engagement features on Twitter, is important for health professionals and others to use, because it may decrease the likelihood of creating “echo chambers,” whereby health professionals may be tweeting only to other health professionals (ie, their followers). Furthermore, it is difficult to knowingly target parents by directly messaging or tweeting to them; however, it is more feasible and realistic to indirectly target parents by creating tweets that parents are more likely to “stumble” across on the basis of searches, key words, or hashtags, or by retweeting parent-focused tweets.

Tweets meant to reach and communicate to parents appeared to be most prominent around awareness days and months, such as National Cancer Awareness Day and Cervical Cancer Awareness Month, and were almost entirely retweets. Leveraging national awareness days to potentially reach the greatest number of followers may be a strategy for health organizations. Furthermore, the potential for a tweet to be a retweet of another organization was much higher during this spike, suggesting the importance of crafting messages that fully use the engagement features of Twitter during this period; many of the retweeted tweets in our sample, from prominent health organizations, did not include links or hashtags.

Although the overall volume of tweets sent by health professionals that were intended for parents was low, the highest number of these tweets also spiked around awareness days and hosted chat events. For example, in January, during Cervical Cancer Awareness Month, a hosted Twitter chat focused on improving girls’ health. Twitter chats have been used by organizations to mobilize a Twitter community to discuss and communicate about a particular topic. This strategy may be effective in connecting health professionals and parents around an important health topic, such as the HPV vaccine, using social media as a platform for communication. This strategy is underused. In our sample, many of the tweets in this spike did not explicitly mention the HPV vaccine, which may have been due to the fact that much of the spike was driven by a hosted chat event that focused on cervical cancer awareness, and the HPV vaccine was mentioned but not discussed during the chat.

Our study has limitations. First, we did not examine tweets sent by parents but rather tweets intended for parents. We framed our analysis this way to focus on a key driver of vaccine uptake: a health professional’s recommendation. Research has documented the strength of this effect on vaccine uptake, and we sought to examine whether this communication behavior was present on Twitter. Although our findings highlighted the positive sentiment of health professionals, most of the communication centered on disseminating new scientific evidence and was not necessarily directed toward a parent audience.

Second, our models for classifying user type, intended audience, and tweet characteristics could have misclassified the variable categories. We developed the classifiers by using an iterative process and checked a subsample of classified tweets; however, because the accuracy of each classifier was not 1.0, some subsets may have been misclassified. To help validate our classification models and study findings, we compared our findings in our full sample of tweets with those of 2 recent studies that used a similar methodology and analytic approach (19,20). We found that the distribution of tweet sentiment was similar across all studies. Of 83,551 and 110,778 tweets and retweets classified in the other studies, 25.1% and 32.0% of the respective samples were coded as negative sentiment (compared with 25.3% of our sample).

Third, although our study examined spikes in communication, we did not examine the nonspike patterns, which tend to be more continuous and durable. We did compare spike characteristics of the subsamples with the spike characteristics of the full sample of tweets; still, future work may consider examining nonspike periods. Our focus on spikes, or increased activity, was informed by studies highlighting the predictive nature of an increase in online communication with offline behaviors, representing an important and specific phenomenon (16).

Disseminating and communicating information on new scientific evidence that supports the HPV vaccine is important to strengthen uptake, and an opportunity exists to better synchronize dissemination of scientific evidence with national awareness days to maximize potential reach and impact. Syncing communication messages with seasonal events is something that the American Academy of Pediatrics has already championed, by producing a toolkit for HPV vaccination messages on Twitter (28). To continue developing strategic communication messages, health professionals may also consider personalizing the scientific evidence by making a clear recommendation to parents or by describing personal experiences or providing opinions that are supported by the new evidence. This strategy is supported by findings that show that the most persuasive messages from physicians to parents are straightforward and strong, such as “your child could get cancer as an adult, but you can stop that right now” (29), as well as messages that take an “announcement” approach that may normalize the vaccine, as compared with a “conversation” approach that may be less effective in mitigating hesitancy (30).

Finally, most research to date has documented the importance of a physician’s recommendation in the context of a clinical encounter, that is, a face-to-face interaction. Although this evidence has been used to inform and tailor the clinical encounter, a gap remains in understanding how a physician’s recommendation contributes to vaccine uptake in a virtual space, particularly in the social media environment. This understanding is important for the Millennial generation that has already begun to transition into parenthood. Norms in health care and health provider interactions will likely shift, and lines between face-to-face and online/virtual interaction will continue to blur. As parents and adolescents continue to use online resources and social media to look for and communicate about health information, health professionals and health organizations have an opportunity to better connect with these audiences by leveraging existing consumer behaviors. By doing so, there is the potential to disseminate important information and evidence in a more strategic way, maximizing reach and impact, with the ultimate goal being higher rates of vaccination.
